# A New Year and a New Volume

**Published:** 1880-01

**Authors:** 


### Editorial.

A New Year and a New Volume.—The present number of the Medical Journal and Examiner marks the commencement of a new year, a new decade and a new volume.

So far as present indications can be interpreted, it also coincides with the commencement of a new era of prosperity in all departments of industry throughout our country, and of comparative health and comfort in all circles of society.

Hence it is an excellent time for all our old subscribers to renew their subscriptions, and for many new ones to send in their names and money, and thereby add the ample pages of the Journal and Examiner to their valuable sources from which to gather new thoughts and new resources to aid them in combatting disease and preserving human health and life. We shall do our best to make it worthy of the patronage of all members of the profession, and close this note by cordially wishing all our readers a *Happy New Year*. D.

